# Effect of Metformin Therapy on Biological Properties and Prognosis of Breast Cancer Patients Complicated with Type-2 Diabetes

**DOI:** 10.12669/pjms.38.5.5135

**Published:** 2022

**Authors:** Xiaolu Yan, Zhe Gao, Yang Li, Qingxia Li, Xinna Deng

**Affiliations:** 1 Xiaolu Yan, Department of Oncology, Hebei General Hospital, Shijiazhuang 050051, Hebei, China; 2 Zhe Gao, Department of Endocrinology and Metabolic Diseases, Hebei General Hospital, Shijiazhuang 050051, Hebei, China; 3 Yang Li, Department of Oncology, Hebei General Hospital, Shijiazhuang 050051, Hebei, China; 4 Qingxia Li, Department of Oncology, Hebei General Hospital, Shijiazhuang 050051, Hebei, China; 5 Xinna Deng, Department of Oncology, Hebei General Hospital, Shijiazhuang 050051, Hebei, China

**Keywords:** Type-2 diabetes, Breast cancer, Metformin, Biological properties, Prognosis

## Abstract

**Objectives:**

To evaluate the effect of Metformin therapy on patients of breast cancer with complications of Type-2 diabetes.

**Methods:**

Altogether 102 cases of breast cancer complicated with Type-2 diabetes admitted into Hebei General Hospital from January 2019 to December 2020 were included in the study. They were divided into two groups per whether Metformin was administered in the regimen, namely Metformin group and non-Metformin group. In the meanwhile, 106 cases of breast cancer without Type-2 diabetes admitted in the same period were selected to form a control group. Three groups were compared in terms of general data (incl. age, body mass, family history, menopause or not), clinical staging, tumor histological differentiation, molecular subtyping (Incl. Luminal A, Luminal B, ERBB2+, Basal-like) and prognosis.

**Results:**

Compared with the control group, Metformin group and non-Metformin group presented more patients with an older age and post-menopause state (*P*<0.05), but the latter two groups were not significantly different (*P* > 0.05). Patients in Metformin group and non-Metformin group had higher clinical staging and histological differentiation and more cases of Basal-like subtype than those in the control group (*P* < 0.05), without significant difference between those two groups (*P* > 0.05). More cases of local relapse, lymphatic and distant metastasis were seen in Metformin and non-Metformin groups, but the differences were not significant (*P* > 0.05). Both groups had lower 5-year survival rates than the control group (*P* < 0.05). Metformin group had a higher overall survival rate as well as a survival rate free of other lethal reasons than the non-Metformin group (*P* < 0.05) but was not significantly different from the control group in the survival rate free of other lethal reasons (*P* > 0.05).

**Conclusions:**

Type-2 diabetes remains one of the risk factors affecting breast cancer development, progress and prognosis, which could lower the 5-year overall survival rate among breast cancer patients. This is especially evident among menopaused women. Metformin therapy may improve the prognosis of patients of breast cancer complicated with Type-2 diabetes.

## INTRODUCTION

The incidence of breast cancer accounts for the top among all female malignant tumors by being 28% or so, severely jeopardizing women’s health.^1^,^2^ On the other hand, Type-2 diabetes has become a public health concern across the world. As speculated in some study, the number of adults suffering from Type-2 diabetes is going to rise from 0.17 billion in 2000 to 0.36 billion by 2030.^3^ Numerous studies have revealed complications in breast cancer patients suffering from diabetes, because 20% of breast cancer patients have been found to have diabetes in the early stage under the effect of such mechanisms as hyperglycemia and hyperinsulinemia.^4^,^5^ It has been testified in studies concerning Type-2 diabetes and malignant tumors that Type-2 diabetes could increase the chance of breast cancer in some patients.^6^

Being effective in improving symptoms of diabetes like hyperglycemia and hyperinsulinemia, Metformin is also found promising in fighting cancer.^7^ Some animal and cellular studies have been conducted to see the antitumor mechanism of Metformin. In their clinical research, Tang^8^ suggested it necessary to determine the effect of Metformin on reducing breast cancer incidence and prognosis through clinical trials based on a systematic review. Therefore, to further explore the relationship between Type-2 diabetes and breast cancer and the effect of metformin treatment on prognosis. We performed a retrospective analysis of 102 breast cancer patients complicated with Type-2 diabetes and 106 breast cancer patients with no complications.

## METHODS

### Ethical Approval:

The study was approved by the Institutional Ethics Committee of Hebei General Hospital on July 08, 2019 (No. K20190423), and written informed consent was obtained from all participants.

This is a retrospective study. The sample size required for each group was calculated by the formula 
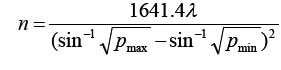
. Altogether 102 cases of breast cancer complicated with Type-2 diabetes admitted into our hospital from Jan. 2019 to Dec. 2020 were collected according to the principle of random draw. They were divided into two groups (Metformin & non-Metformin) as per whether Metformin was included in their therapeutic regime. Another 106 cases of breast cancer alone were also included through random sampling to form a control group. All the breast cancer patients underwent surgical operation and were pathologically confirmed as having invasive breast cancer after the surgery.

### Inclusion criteria:

*Diabetes diagnosis criteria:* The diagnostic criteria were based on the 1999 WHO/IDF diagnostic criteria, namely symptoms + fasting plasma glucose ≥ 7.0 mmol/L, or random blood glucose ≥ 11.11 mmol/L. *Diagnosis of breast cancer*: All breast cancer patients were diagnosed by ultrasound and MRI, and confirmed as invasive carcinoma by surgical histopathological examination. The age ranged from 45 to 65 years old. Clinical staging of breast cancer at I-III. Histological grading of breast cancer was 1-3. The duration of breast cancer was less than one year. Patients or their family members signed consent Form..

### Exclusion Criteria:

- Patients of type-1 diabetes or secondary diabetes.
- Patients of breast carcinoma in situ, bilateral breast cancer, or male breast cancer.
- Patients suffering from severe heart, liver and kidney insufficiency and unable to be controlled satisfactorily.
- Patients not suitable for metformin treatment.
- Patients who need insulin to control their blood glucose.

### Research methods:

Demographical data of patients in three groups were collected to compare them in terms of their clinical characteristics (incl. age, body mass, family history, menopause or not), pathological features and prognosis. The clinical staging, histological grading and breast cancer’s molecular subtyping were done in accordance with American Joint Committee on Cancer (AJCC, 7^th^ Edition), Scarff-Bloom-Richardson (SBR) improved grading system and St. Gallen Breast Cancer Conference held in March 2011, respectively. Blood glucose control was implemented, both before and after the surgical operation. Metformin was given at doses of 500mg twice or three times a day, adjusted according to blood glucose results. The adverse reactions such as lactic acidosis, gastrointestinal reaction, hypoglycemia and renal insufficiency were observed during medication, and the hypoglycemic program and symptomatic treatment was adjusted. All patients in the study were followed up by telephone for five years after receiving treatment at our hospital.

### Statistical Process:

Data were processed using SPSS 22.0 statistical software. All measurement data were expressed as mean ± standard deviation, and all enumeration data were checked with *χ*^2^ test or rank sum test. *P* < 0.05 was considered significant.

## RESULTS

There were no significant differences in body mass index or family history among the three groups (*P* > 0.05). The proportion of elderly patients and menopausal patients in the metformin and non-metformin groups was significantly higher than that in the control group (*P* < 0.05), but there was no significant difference between the former two groups (*P* > 0.05) (Table-I).

**Table I:** A comparison of three groups in clinical features [χ¯±s,*n*(%)].

Group	No.	Age (year)	Body mass (Kg/m²)	Family history [*n*(%)]	Menopause or not [*n*(%)]	

					Menopause	Non-Menopause
Metformin	44	56.78±7.95	26.27±8.22	8(18.2)	15(34.1)	29(65.9)
Non-Metformin	58	55.67±8.28	27.96±3.75	12(20.7)	18(31.0)	40(69.0)
Control	106	50.48±9.16	26.85±3.42	18 (17.0)	46(43.4)	60(56.6)

The proportion of patients with grade I clinical stage was significantly lower in the Metformin and non-Metformin groups than in the control group, while the proportion of patients with Grade- II or III clinical stage was significantly higher (*P*<0.05). The proportion of patients with Grade- I or II histological grading was significantly lower in the Metformin and non-Metformin groups than in the control group, while the proportion of patients with grade 3 histological grading was significantly higher than the control group (*P*<0.05). Compared with the control group, Metformin and non-Metformin groups had a higher proportion of patients with molecular subtypes Luminal A or Basal-like, and a lower proportion of patients with molecular subtypes Luminal B or ERBB2+ (*P*<0.05). These results indicate that patients of breast cancer complicated with Type-2 diabetes may have worse prognosis. When Metformin group and non-Metformin group were compared in those three aspects, they weren’t significantly different (*P* > 0.05) (Table-II, III & IV).

**Table II:** A comparison of three groups in clinical staging [*n*(%)].

Group	No.	Clinical staging			

		I	II	III	IV
Metformin	44	12 (27.3)	17(38.6)	15(34.1)	0(0)
Non-Metformin	58	14(24.2)	22(37.9)	22(37.9)	0(0)
Control	106	47(44.3)	34(32.1)	25(23.6)	0(0)

**Table III:** A comparison of three groups in histological grading [n (%)].

Group	Case	Histological grading		

		1	2	3
Metformin	44	26(59.1)	11(25.0)	7(15.9)
Nom-Metformin	58	34(58.6)	13(22.4)	11(19.0)
Control	106	65(61.3)	32(30.2)	9(8.5)

**Table IV:** A comparison of three groups in molecular subtyping [n (%)].

Group	No.	Molecular subtyping			

		Luminal A	Luminal B	ERBB2+	Basal-like
Metformin	44	10(22.7)	16(36.4)	3(6.8)	15(34.1)
Non-Metformin	58	11(19.0)	22(37.9)	5(8.6)	20(34.5)
Control	106	14(13.2)	54(51.0)	13(12.2)	25(23.6)

Compared with patients in the control group, those in Metformin group and non-Metformin group suffered from higher risks of local relapse, lymphatic and distant metastasis, but the differences all remained insignificant (*P* > 0.05). The latter two groups had significantly lower 5-year overall survival rates than the control group (*P* < 0.05), while they weren’t evidently different from each other when compared alone in those aspects (*P* > 0.05). The Metformin group had significantly higher overall survival rate and survival rate free of other lethal reasons than the non-Metformin group (*P* < 0.05), but it was not significantly different from the control group (*P* > 0.05) (Table-V).

**Table V:** A comparison of three groups in prognosis.

Group	No.	Breast cancer incident (no.)			Lethal cause (no.)		Survival (no, %)	

		Local relapse	Distant metastasis	Lymphatic metastasis	Breast cancer	Others	Overall survival rate	Survival rate free of other lethal reasons
Metformin	44	4	6	5	5	3	36(81.8)	39(88.6)
Non-Metformin	58	6	11	7	10	4	44(75.9)	48(82.8)
Control	106	7	12	11	15	4	91(85.8)	95(89.6)

## DISCUSSION

In recent years, breast cancer and diabetes have become common diseases threatening human health and life. Their morbidities and mortalities are also increasing. Both domestic and foreign studies have found that the incidence of malignant tumors in patients with diabetes was significantly increased. Breast cancer has become the most common malignant tumor and important cause of death in women.^1^,^9^ The connection between diabetes and breast cancer was gradually attached with great importance. Type-2 diabetes may be a risky factor affecting the incidence of breast cancer.^10^ Breast cancer is heterogeneous. According to its clinical characteristics and molecular typing, the treatment response and prognosis are also different.^11^ For this reason, we examined the clinical characteristics, pathological features and prognosis of patients in three groups under question.

In this study, there was no significant difference in body weight among the three groups, which indicating obesity is a risk factor for both diabetes and breast cancer. Evidence-based medicine has confirmed that overweight and obesity could enhance the probability of breast cancer for women in menopause.^12^ The pathogenesis may stay linked to more in vivo adipokines, change in the levels of hormones like estrogen, insulin, and leptin, biological properties arising from obesity and chronic inflammation as well.^13^ Li, et al. reported higher ratios of older age and menopause state in the patients of breast cancer with Type-2 diabetes complications.^14^ Michels discovered the probability for Type-2 diabetes patients to get breast cancer was 17% higher than those non-diabetes patients and this was especially evident in the menopaused women.^15^ The aforesaid academic results were in line with our study findings here that include an older average age and a higher ratio of menopause (65.9% and 69%, respectively) in the Metformin group and non-Metformin group than the control group (56.6%). However, no statistical difference is detected in baseline clinical features between Metformin group and non-Metformin group.

As for pathological features, according to the findings made by Peairs^16^, patients of breast cancer with Type-2 diabetes complications had more advanced clinical staging and worse prognosis. As shown in this study, both Metformin group and non-Metformin group had more advanced clinical staging than the control group, since 72.7% and 75.8% of patients in those two groups were in Stage II/III in contrast to 55.7% in the control group, which was consistent with the previous studies. In addition, molecular subtyping also affected the prognosis of patients. Patients of triple negative breast cancer (TNBC) were usually expected to experience the worst prognosis. In our study, histological grading was higher and Basal-like molecular subtype (or TNBC) was more common in Metformin group and non-Metformin group, which was similar to the literature.^16^ However, two groups of breast cancer with Type-2 diabetes complications were not significantly different from each other in terms of clinical staging, histological grading and molecular subtyping. In this study, more patients in Metformin group experienced local relapse and lymphatic and distant metastasis than those in the non-Metformin group though in a significant way. Type-2 diabetes patients experienced a lower 5-year overall survival rate than normal people, no matter whether Metformin was administered. As indicated by the perspective study of Villareal^17^ on recurrent and metastatic breast cancer patients, compared with normal glucose group, the high glucose group (> 130 mg/dl) had a significantly shorter overall survival period. Erickson^18^ also witnessed worse prognosis and lower overall survival rate among female breast cancer patients that had glycosylated hemoglobin >7% when compared with the control group. All the findings listed above suggested that abnormal glucose metabolism may speed up the progress of breast cancer. Thus, it is important to enhance control on disturbance of carbohydrate metabolism among breast cancer patients.

Metformin is a first-line oral hypoglycemic agent for treating Type-2 diabetes, which inhibit gluconeogenesis in livers via strengthening hepatic sensitivity to insulin so as to bring down the levels of insulin and glucose in blood circulation. Thus, we divided Type-2 diabetes patients into two groups as per their hypoglycemic therapy for prognosis analysis, namely Metformin group and non-Metformin group. Metformin group had higher overall survival rate than the non-Metformin one, even after other lethal causes were ruled out. But no statistical difference was detected between them and the control group. Reduced overall survival rate observed in the non-Metformin group may be partly attributed to non-cancer factors^19^, such as untimely or non-standard treatment and death arising from diabetic complications in varying degrees. A conclusion that was worth of special mention here was that even after other lethal reasons were ruled out, Metformin group and non-Metformin group enjoyed obvious survival strengths. This further showed potential of Metformin in fighting cancer. It may be used as a brand-new anti-cancer agent in clinical practice. Studies indicated Metformin was effective in improving glycometabolism and fighting multiple cancers. The study of Kusturica^20^ indicated patients of Type-2 diabetes had lower risk of having breast cancer when taking Metformin than other hypoglycemic agents. When conducting a retrospective study on breast cancer patients that accepted adjuvant chemotherapy, El-Benhawy^21^ founded administration of Metformin during adjuvant therapy period could significantly improve the survival rate and overall survival rate of patients of breast cancer complicated with Type-2 diabetes. Bowker et al followed 10,309 cases of tumor complicated with Type-2 diabetes for 5.5 years on average, finding fatality rates among patients taking Metformin, sulfonylureas and insulin to be 3.5%, 4.9% and 5.8%, respectively. This cohort study proved tumor patients having Type-2 diabetes had lower fatality rate if taking Metformin.^22^ The specific antitumor mechanism of Metformin needs further elucidation. As reported, the drug fights cancer mainly via reducing the glucose and insulin in blood circulation.^23^ Besides, Metformin could also inhibit mTOR (Mammalian Target of Rapamycin) compound 1 signal pathway through liver kinase B1-mediated AMPK (AMP-activated protein kinase) activation so as to inhibit cell growth.^24^ According to the study of Schott, Metformin could improve antitumor immunity by stimulating Tm lymphocyte proliferation of breast cancer patients.^25^ The drug could selectively inhibit breast cancer stem cells^26^ as well as the expression of adhesion molecule CD24,^27^ which was both a marker of breast cancer stem cells and an indicator of TNBC’s poor prognosis. Nevertheless, the breast cancer cell inhibiting mechanism of Metformin may shed light on seeking new therapeutic targets and affect the decision-making concerning clinical mediation. All those have demonstrated the clinical research value of Metformin in breast cancer prevention and treatment.

### Limitations of this study:

This was a retrospective study. It is necessary to further conduct a prospective long-term observational study on the preventive effect of Metformin in which actual prescription period and dosage over the time should be analyzed. The study should also include non-diabetes patients in order to determine whether Metformin is effective only for the breast cancer patients with diabetes complications or all the breast cancer patients. In future studies we should further determine the acting mechanism and clinically suitable population of the Metformin.

## CONCLUSION

Diabetes could be a risk factor affecting breast cancer development and prognosis and reducing the 5-year overall survival among breast cancer patients. This was especially prominent in the menopaused women. Special attention should be paid to the glucose control during treatment of breast cancer patients complicated with Type-2 diabetes. Administration of Metformin could improve the survival rate and prognosis of breast cancer patients complicated with diabetes regardless of its unclear antitumor mechanism.

### Authors’ Contributions:

**XY & ZG:** Designed this study and prepared this manuscript. They are also responsible for the accuracy and integrity of this study.

**YL & QL:** Collected and analyzed clinical data.

**XD:** Significantly revised this manuscript.
